# A Research Agenda to Underpin Malaria Eradication

**DOI:** 10.1371/journal.pmed.1000406

**Published:** 2011-01-25

**Authors:** Pedro L. Alonso, Graham Brown, Myriam Arevalo-Herrera, Fred Binka, Chetan Chitnis, Frank Collins, Ogobara K. Doumbo, Brian Greenwood, B. Fenton Hall, Myron M. Levine, Kamini Mendis, Robert D. Newman, Christopher V. Plowe, Mario Henry Rodríguez, Robert Sinden, Laurence Slutsker, Marcel Tanner

**Affiliations:** 1 Barcelona Centre for International Health Research (Hospital Clínic, Universitat de Barcelona), Barcelona, Spain; 2 Centro de Investigaçao em Saude da Manhiça, Maputo, Mozambique; 3 Nossal Institute for Global Health, University of Melbourne, Melbourne, Australia; 4 Immunology Institute-Universidad del Valle, Cali, Colombia; 5 Centro de Investigación Caucaseco, Cali, Colombia; 6 School of Public Health, University of Ghana, Accra, Ghana; 7 International Center for Genetic Engineering and Biotechnology, Delhi, India; 8 University of Notre Dame, Notre Dame, Indiana, United States of America; 9 University of Bamako, Bamako, Mali; 10 London School of Hygiene & Tropical Medicine, London, United Kingdom; 11 National Institute of Allergy and Infectious Diseases (NIAID), Baltimore, Maryland, United States of America; 12 Center for Vaccine Development, University of Maryland, Baltimore, Maryland, United States of America; 13 Global Malaria Program, World Health Organization, Geneva, Switzerland; 14 Howard Hughes Medical Institute/University of Maryland School of Medicine, Baltimore, Maryland, United States of America; 15 Instituto Nacional de Salud Pública, Cuernavaca, Mexico; 16 Imperial College, London, United Kingdom; 17 US Centers for Disease Control and Prevention, Atlanta, Georgia, United States of America; 18 Swiss Tropical and Public Health Institute and University of Basel, Basel, Switzerland

## Abstract

The interruption of malaria transmission worldwide is one of the greatest challenges for international health and development communities. The current expert view suggests that, by aggressively scaling up control with currently available tools and strategies, much greater gains could be achieved against malaria, including elimination from a number of countries and regions; however, even with maximal effort we will fall short of global eradication. The Malaria Eradication Research Agenda (malERA) complements the current research agenda—primarily directed towards reducing morbidity and mortality—with one that aims to identify key knowledge gaps and define the strategies and tools that will result in reducing the basic reproduction rate to less than 1, with the ultimate aim of eradication of the parasite from the human population. Sustained commitment from local communities, civil society, policy leaders, and the scientific community, together with a massive effort to build a strong base of researchers from the endemic areas will be critical factors in the success of this new agenda.

## Abstract 2

Pedro Alonso and colleagues introduce the Malaria Eradication Research Agenda (malERA) initiative and the set of articles published in this *PLoS Medicine* Supplement that distill the research questions key to malaria eradication.

**Summary Points**

- Malaria remains a major global public health problem, but a recent paradigm shift has moved the emphasis from control of malaria to the interruption of malaria transmission and ultimately malaria eradication
- The Malaria Eradication Research Agenda (malERA) initiative was convened in 2008 to define the knowledge base, strategies, and tools required to eradicate malaria from the human population
- A two-year consultative process has resulted in the preparation of a detailed research and development agenda for malaria eradication, which is reported in this Supplement
- Implementation of this research agenda might enable the elimination of malaria, even in the most difficult areas
- However, to achieve the aim of malaria eradication in a timely manner, commitment to implementing this agenda must begin immediately

## Introduction

The unacceptable health burden of malaria, and its economic and social impacts on development, have made it a focal point of the international development agenda, and the world has embraced an ambitious plan for scaling up malaria control that progresses towards country-by-country and regional elimination and the ultimate goal of global eradication [1]. Over the past decade, resources and control efforts have intensified to a level not seen since the early days of the World Health Organization's Global Malaria Eradication Program (GMEP) in the late 1950s. Nonetheless, in 2009, with 3.28 billion people living in areas that have some risk of malaria transmission and about 1.2 billion people (one-fifth of the world's population) living in areas with a high risk of transmission (more than one reported case per 1,000 population per year), there were about 225 million cases of clinical malaria and 781,000 malaria-related deaths. Today, there is ongoing malaria transmission in 106 countries. Eighty-one of these countries are focusing on control, while 25 are in pre-elimination, elimination, and prevention of reintroduction phases; Morocco, the United Arab Emirates, and Turkmenistan have recently been certified as malaria free [2]–[4].

These statistics emphasize the direness of the current malaria burden but also benchmark the accomplishments and progress that have been achieved in malaria control. Following declarations at the Malaria Forum in October 2007 convened by the Bill & Melinda Gates Foundation, and subsequent support voiced by the World Health Organization (WHO), the Roll Back Malaria (RBM) Partnership, and many other organizations and institutions, the paradigm of malaria control and elimination has been extended to encompass an ultimate goal of malaria eradication [1],[2],[5]. The question is no longer whether international agencies and national health authorities should be mobilized to pursue the goal of malaria eradication, but rather when and how.

A key question, however, is whether elimination from all regions of the world (eradication) is feasible with the current tools and state of knowledge. For a number of reasons, we believe that the answer is “no.” First, malaria is not a single disease. The five *Plasmodium* species *(falciparum, vivax, ovale, malariae, knowlesi)* that cause human malaria are transmitted by more than 30 Anopheline mosquito species with diverse breeding and feeding habits, and result in different disease spectra in different population target groups and epidemiological settings. Second, current malaria control and elimination programs face remarkable heterogeneity of transmission dynamics of malaria in endemic areas, including differences in parasite, vector, human, social, and environmental factors. Third, operational limitations include underperforming health services, lack of political will, insufficient financial, social and human resources, and for some areas, inadequate tools to interrupt transmission given an exceedingly high force of transmission. Each country presents different combinations of these problems and their determinants. Thus, a widely held view suggests that with currently available tools, much greater gains could be achieved, including elimination from a number of countries and regions, but that even with maximal effort we will fall short of elimination in many areas and of global eradication [6]. For definitions of terms used regarding malaria eradication see Box 1.

**Box 1. Clarifying the Goals and Definitions**

- **Control:** Reduction of disease incidence, prevalence, morbidity, or mortality to a locally acceptable level as a result of deliberate efforts; continued intervention measures are required to maintain the reduction.
- **Elimination:** Reduction to zero of the incidence of locally transmitted malaria infection in a defined geographical area as a result of deliberate efforts; continued intervention measures are required to prevent reestablishment of transmission.
- **Eradication:** Permanent reduction to zero of the global incidence of malaria as a result of deliberate efforts; intervention measures are no longer needed [1].
- **What species?** Although the eradication of *P. falciparum*, the most serious form of malaria, would constitute an historic public health achievement, the coexistence of transmission of *P. falciparum* and *P. vivax* in many areas of the world together with the fact that they are the species responsible for the major burden of disease, make it necessary to aim for the eradication of both.

## Mixed Success and Failure of Past Malaria Control and Elimination Efforts

A detailed discussion of all the factors involved in the partial success of the past eradication campaign is beyond the scope of this introduction, but three critical elements can be highlighted. First, there was insufficient recognition of the heterogeneity of malaria transmission and disease. Much of the optimism that inspired WHO GMEP in 1955 was based on the successful outcomes of earlier control programs that benefited from a combination of biological, parasitological, social, and environmental factors that favoured success (e.g., the rarity of DDT-resistant Anophelines and of chloroquine-resistant parasites). Second, the first WHO GMEP (1955-1969) was predicated on an assumption that the available knowledge and tools were sufficient to achieve worldwide eradication. A single strategy that would work everywhere—“one size fits all”—proved to be ill-founded because it underestimated the challenges of dealing with the extremely efficient vectors in Africa (*An. gambiae)* and with transmission by outdoor-feeding mosquitoes that were not susceptible to attack by indoor residual insecticide. It also did not allow for the lack of safe drugs for mass administration to remove all infectious parasites from symptomatic and asymptomatic carriers, particularly from people carrying *P. vivax* or *P. ovale,* species that establish latent liver infections that are responsible for relapses months or years following initial infection. Third, insufficient research in biomedical and social sciences and inadequate local application of research findings across a wide variety of settings are widely viewed to have contributed to demoralization and waning effort when tools proved ineffective or could not be adequately implemented. The neglect of malaria research during and after the campaign did long-term damage. These elements resulted in a lack of progress that in turn compromised continued financial support [7].

## Current Malaria Control Efforts: The Goal of Eradication and Its Research and Development Implications

**Figure 1:**
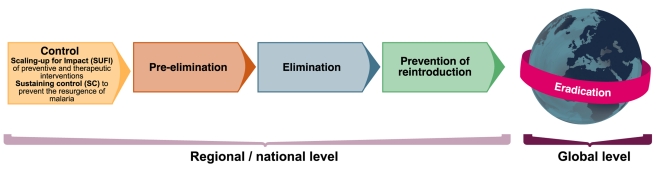
Epidemiological milestones [**1**],[23****]. Image credit: Fusión Creativa.

The past decade has witnessed renewed investment in malaria control and substantial increases in funding for malaria research. The Roll Back Malaria Global Malaria Action Plan (GMAP) and WHO have recently revised and updated the strategy and the steps for scaling up and sustaining malaria control (Figure 1). In addition, the Malaria Elimination Group (MEG), a group of scientists, public health decision makers, control program managers, and funders, has compiled a guide to policy makers for areas that embark or have embarked on elimination strategies [8].

Reductions in disease incidence are being documented, even in some areas of sub-Saharan Africa that constitute the heartland of malaria transmission [2]. There are, however, significant threats to current progress that cannot be ignored, and unmet needs that will continue to be central to the global research agenda for improving malaria control and eventually achieving eradication. Notable examples are the emergence of artemisinin resistance and the consequent need for improved strategies to contain dissemination of resistant parasite strains coupled with accelerated research into potential new drugs for first-line treatment [9],[10]. Similarly, new insecticides are urgently needed to replace those threatened by increased mosquito resistance [11], and accelerated development of vaccines that can impact on malaria incidence, disease, and death remains a high priority [12].

Complementing the current research agenda—primarily directed towards improving malaria control and reducing morbidity and mortality—with research on developing tools, interventions, and strategies to interrupt transmission and ultimate eradication of the parasite from the human population constitutes a true paradigm shift.

## The malERA Initiative

To catalyze this paradigm shift towards malaria elimination and eradication, it was necessary to design a process to bring together the best scientific minds in the malaria community. That process is the Malaria Eradication Research Agenda (malERA) initiative, which was established to complement GMAP and which aims to define the critical knowledge base, strategies, and tools required to reduce the basic reproduction rate (*R*_0_ or the number of secondary cases arising from a single case) to less than one.

**Figure 2:**
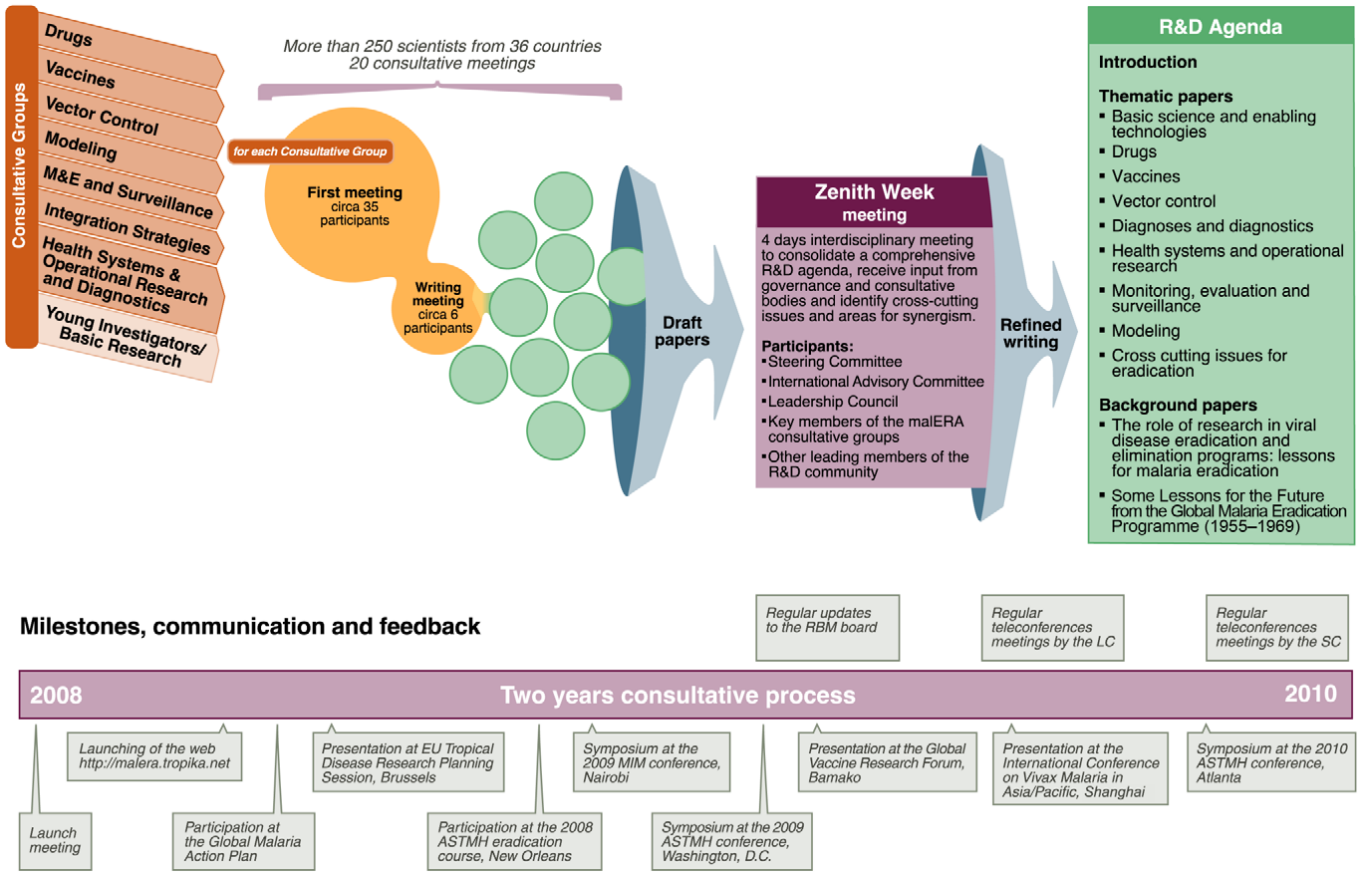
Consultative process towards a consolidated research and development agenda for malaria eradication. Image credit: Fusión Creativa.

**Figure 3:**
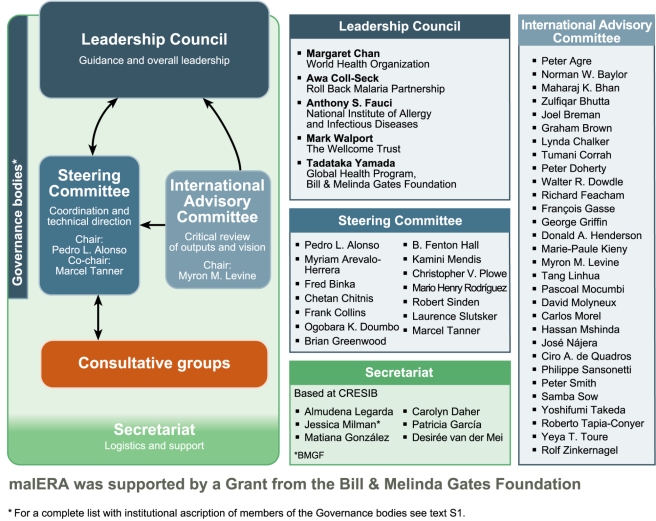
The malERA governance bodies. Image credit: Fusión Creativa.

Scientists involved in malaria research were challenged to develop a multidisciplinary, global research and development agenda that would be actionable by research and public health agencies and funders/sponsors and available for discussion and debate through publication in a readily accessible format. The process engaged more than 250 scientists in a series of 20 consultations around the world (Figure 2) and was managed by a three-tier governance structure (Figure 3). The rest of this article briefly introduces the work undertaken by the various malERA Consultative Groups and presented in the other articles in this Supplement.

## Tools to Interrupt Malaria Transmission

To reduce the basic reproduction rate to less than 1, and hence to interrupt transmission, interventions are needed to reduce the reservoir of infection, the time that a person or a mosquito is infectious, and the rate at which infections are spread. This goal can be achieved by drugs or vaccines directed against the parasite or by new tools that attack the vector, with the support of improved diagnostics and surveillance.

### Drugs: Single Encounter Radical Cure and Prophylaxis

In the recent past, drug development efforts were guided by the need for first-line drugs to treat *P. falciparum* infections with an increasing emphasis on drugs with a short half-life that potentially minimize the risk of development of resistance rather than on drugs with a long half-life that have benefits for dosing and post-treatment prophylaxis [13]. Treatment of infected individuals with a variety of drug regiments has been used successfully in combination with intensive vector control to eliminate malaria from areas with relatively strong health systems and stable populations. However, interruption of malaria transmission is likely to require a new set of drugs and formulations.

As described in more detail in the article by the malERA Consultative Group on Drugs [14], such drugs will need to be used both in stable transmission areas and in complex urban or remote rural areas, with poorly functioning health systems where concerted campaigns may be the only way of achieving high coverage or preventing reintroduction by migrants or travelers from endemic regions. For such campaigns to impact effectively on inaccessible populations, a single encounter between health providers and target populations is critical. Single Encounter Radical Cure and Prophylaxis (SERCaP) has a target product profile (TPP) that includes radical cure, defined as elimination of all parasites (including the long-lived hypnozoites of *P. vivax* or *P. ovale* in the liver), suitability for mass administration (including administration to healthy subjects and the consequent need of a very good safety profile), and prophylaxis for at least 1 month after treatment, to outlast the typical development period of *Plasmodia* parasites in Anopheline mosquitoes. A drug with this profile would perform in a similar way to a highly efficacious pre-erythrocytic (infection-preventing) vaccine.

A drug with this TPP may take a long time to develop, but the development of new drugs that meet some of these essential requirements could dramatically improve chances of eradication. For example, development of new safe and effective drugs that block the infectivity of the mature sexual forms of *P. falciparum* gametocytes and/or the dormant hepatic forms (hypnozoites) of *P. vivax* could have a profound impact on transmission rates and would be valuable tools in the efforts to contain and eliminate parasite strains resistant to first-line treatment drugs. Presently, only the 8-aminoquinolines are known to be effective against both *P. vivax* hypnozoites and *P. falciparum* stage-five gametocytes. Unfortunately this class of drugs has significant side-effects in some individuals, particularly hemolysis in those with G6PD deficiency, that compromise their widespread use in mass administration for elimination [14].

### Vaccines that Interrupt Malaria Transmission

Vaccines currently in clinical development have the primary aim of reducing morbidity and mortality from *P. falciparum* in young children living in highly endemic countries. However, with the new goal of elimination and eradication, vaccines that will reduce and contribute to interruption of transmission also need to be developed. The broader concept of “vaccines that interrupt malaria transmission (VIMT)” is introduced by the malERA Consultative Group on Vaccines to replace the term “transmission blocking vaccines” (TBVs), which has been used widely to refer to vaccines that target only the sexual and mosquito stages of the parasite [15]. VIMT could include antivector vaccines that target mosquito molecules essential for parasite development, highly effective pre-erythrocytic or erythrocytic stage vaccines, and vaccines targeting parasite antigens of sexual and mosquito stages of the infection. The desired TPP identified by the Consultative Group for VIMT indicates that they should be effective against both *P. falciparum* and *P. vivax,* suitable for administration to all age groups, and should impact transmission. Other issues discussed by the group in their article include the need for validated functional assays that measure the reduction in infectivity at the individual level after vaccination that could be used as surrogate measures to predict reductions in transmission rates at the community level. Such surrogate measures will be critical components of a regulatory pathway leading to licensure. Standardized, specific and sensitive methods for assessment of transmission rates, particularly when intensity is low, will be critical in the assessment of vaccine efficacy in interrupting transmission following large-scale deployment of vaccination as an elimination tool [15],[16].

### Vector Control

The overarching goal of vector control is to reduce the vectorial capacity of local vector populations below the critical threshold to prevent ongoing or epidemic transmission. Because it takes a relatively long time (days) after ingestion for *Plasmodia* to become infective to humans in its *Anopheles* vectors, the most effective vector control strategies currently in use rely on interventions like indoor residual insecticide spraying and insecticide treated bednets (ITNs) that reduce vector daily survival rates [17].

The malERA Consultative Group on Vector Control identifies three critical challenges in its article [18]. The most pressing challenge is the development of a coherent research agenda for discovering and developing a broader range of insecticides, with novel modes of action that can circumvent emerging resistance to existing insecticides, in particular, pyrethroid-based insecticides [11]. The second challenge is the development of interventions that affect vectors that do not rest or feed indoors and are therefore not susceptible to current tools. The final critical challenge is the development of novel approaches that permanently reduce the high vectorial capacities of the dominant malaria vectors in sub-Saharan Africa. Genetic control programs based on permanent reduction of the vectorial capacities of natural vector populations have received the most attention to date [19],[20], but the Consultative Group also considers the development of other novel approaches [18].

### Diagnostics

Methods for measuring transmission are central to an elimination agenda. Current methods for measuring transmission that may be applied in endemic areas are time-consuming, expensive, and too insensitive for use in conditions of low and nonuniform infection [21],[22]. Some years after regional elimination, as immunity declines, infection is likely to be symptomatic and may become the best marker of resumed transmission. However, during the early elimination phase in regions previously experiencing high transmission, populations will retain clinical immunity and will not experience symptomatic disease with every infection [23]. Thus, the main challenge identified by the malERA Consultative Group on Diagnoses and Diagnostics and discussed in detail in their article and in the article on Cross-cutting Issues for Eradication [24],[25] is to find a robust, sensitive, and specific standardized method for assessment of transmission intensity in the intervening period when transmission continues at low and nonrandom levels. Improved serological tests have been suggested [26], but other minimally invasive biomarkers could be considered. This information will be essential for modeling potential effects of various interventions alone, or in combination, and for assessing efficacy of transmission–reducing vaccines and drugs. Other challenges for diagnostics discussed by the Consultative Group include the need for tools that can rapidly detect and monitor unexpectedly high transmission that leads to outbreaks and that can identify reintroduction of infections that may be asymptomatic [16],[24].

## Beyond the Tools: Supporting Strategies and the Knowledge Base

### Modeling and Harmonized Data Systems

Substantial advances have been made recently in computational approaches for modeling malaria epidemiology and in model-based approaches to economic evaluation [27]–[29]. As discussed by the malERA Consultative Group on Modeling [16], a significant research challenge for malaria eradication will be to integrate these new approaches into the planning of elimination, surveillance, monitoring, and evaluation, and to create appropriate interfaces for different user communities, including researchers, global and national policy makers, and local-level planners. Modeling can inform the definition of TPPs for new tools and intervention strategies and will be needed throughout a global eradication campaign to analyze the likely effects on malaria and of various elimination strategies and the costs of these strategies [30].

Importantly, a single unifying model will be insufficient to meet all these needs, so multiple modeling efforts need to be coordinated and made accessible to everyone. This harmonization and validation process will require close, iterative collaboration between software engineers, researchers, and malariologists to develop the necessary computer systems and connectivity (cyberinfrastructure). It will also necessitate the creation and maintenance of properly annotated and accessible malariometric databases that include all the research results needed to insert parameters into the models and the model outputs. How this can be achieved is considered in detail by the Consultative Group in their article [16].

### Enabling Technologies and Platforms

The development of new tools for elimination is critically dependent on a vibrant and coherent agenda for basic sciences. We believe there are at least two potentially transformative developments that need to be pursued. First, continuous laboratory culture of *P. vivax*, *P. ovale*, and *P. malariae* needs to be developed to provide an essential platform for studying the biology of the liver stages and sexual forms of these parasites. These forms could be important targets of intervention strategies with drugs, vaccines, or other biological or chemical agents aimed at interrupting transmission. Second, systems analyses of transcription, proteome, and metabolome libraries, rapid screening of drug libraries, high-throughput approaches to antigen identification, and the functional definition of gene products are all feasible but not yet fully exploited, but would bring important new tools to the bench scientist and to field operations. These and other aspects of enabling technologies and platforms are considered in detail in the articles prepared by the malERA Consultative Groups on Basic Science and Enabling Technologies and on Cross-cutting Issues for Eradication [25],[31].

## Health Systems Integration, Operational Research, and Effectiveness-Decay Analysis

The previous formal attempt at global eradication of malaria (1955–1969) depended largely on vertical operations that often bypassed health systems and their health services because it was assumed that eradication operations could be run most efficiently in this way. Many of the elimination efforts failed, because the health systems failed, leading to a pessimistic view that malaria can only be eliminated where economic progress, governance, and efficient health systems are in place to support maintenance of conditions necessary to block transmission [32],[33].

It is now clear that the long-term solution to malaria elimination and eradication will require a systems approach in which malaria-specific interventions and actions are integrated into existing health systems [34]. To achieve this, research is needed into health systems, their readiness to optimize novel programs, systems, tests, or other interventions, and their continuing performance [35]–[37]. During their deliberations, the malERA Consultative Group on Health Systems and Operational Research identified the need for a substantial research approach to establish and validate a tool kit that allows effectiveness-decay analysis of health system impediments to effective and equitable coverage of malaria interventions and that allows decisions to be made on the degree of possible integration of interventions into an existing health system [16],[38]. A further critical component of the research agenda identified by this Consultative Group is the development and validation of a decision-making framework to guide the move from control to elimination.

Finally, but equally importantly, the article by the malERA Consultative Group on Monitoring, Evaluation, and Surveillance considers the need to investigate the performance of surveillance, monitoring, and evaluation by new and old technologies [39],[40] and to evaluate optimal strategies for implementation of surveillance as an active responsive intervention to further reduce transmission [41].

## Training

The last time the world community tried to eliminate malaria, so the joke goes, the only thing that was eliminated was malariologists. For a renewed malaria eradication campaign to have a chance to succeed, it will be essential to train the malariologists and scientists in the multiple disciplines needed for an eradication campaign that might last 50 years, especially in endemic countries. This need cannot be overemphasized. The malaria research community remains small and often dominated by the views and strategies of scientists who sit far away from the problems. A massive effort to train, empower, and sustain research capacity in the endemic countries will be a critical factor for the success of improved control efforts and for the ultimate elimination and eradication of malaria.

## Concluding Remarks

**Figure 4:**
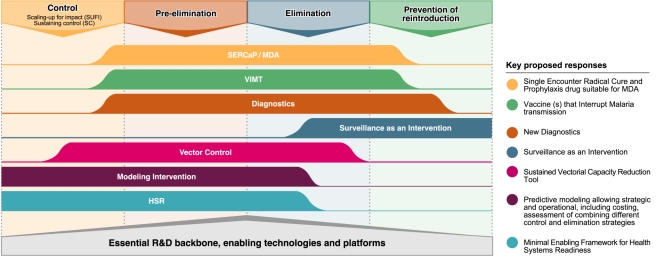
Key research and development issues and their position in relation to the different epidemiological phases towards eradication. Image credit: Fusión Creativa.

The past 2 years have reinvigorated an old malaria paradigm in which reduction of transmission is the driving strategy for malaria interventions. The malaria community has now used the malERA process to propose a research and development agenda that will be essential for regional elimination and eventual global eradication of malaria. Not every tool or strategy considered by the malERA Consultative Groups (see Box 2) will be essential in every situation (see Figure 4), but the complexity and heterogeneity, and in some places, the sheer intensity of transmission, demand that we start without delay to prepare for the most difficult challenges. This focus on the end goal of eradication must not displace our determination and efforts to continue to scale up ongoing efforts for control and to include a research agenda for reducing morbidity in areas of continuing moderate or high transmission. Rather, it must encourage us to supplement our efforts with a structured agenda that can realize the ultimate goal of eradication envisaged by the Global Malaria Action Plan and the Roll Back Malaria Partnership. An important lesson we can learn from other disease elimination efforts is that complacency is dangerous. The parasite and the vector are always evolving, and the human environment is always changing. Thus, new research questions will continually arise during the course of elimination [42], and active malaria research, particularly on the development of new tools, must continue up to the point when eradication is finally achieved. We anticipate that the results of research efforts proposed by our Consultative Groups for each stage of progression, from scaling up for improved control to the elimination phases, will have great synergy in design and application.

**Box 2. Key Examples of Critical Research Needed to Support Elimination and Eradication of *Plasmodium falciparum* and *Plasmodium vivax***

- In vitro culture and study of hypnozoites (persistent liver stages) of *P. vivax*
- Drugs to be used for mass drug administration to clear infections and provide prophylaxis to prevent new infections
- Vaccines that target different stages of the parasite life cycle, or the mosquito, with the key goal of interrupting transmission
- New vector control approaches for (i) outdoor biting/resting mosquitoes and (ii) achieving permanent reductions of vectorial capacity in areas where transmission is predominantly due to the highly efficient vector *A. gambiae*
- New approaches for fast and accurate assessment of transmission at community level
- When to press the elimination button? Tool kits to scientifically determine “health system readiness” for a switch to elimination efforts
- New collaborative approaches to use of mathematical modeling to inform TPPs, and expected outcomes of mixes of intervention
- Strengthened monitoring and evaluation tools and strategies for interrupting transmission that are linked and embedded in the health and social systems

Past efforts at disease eradication, successful or otherwise, have highlighted the importance of sustained commitment from local communities, civil society, policy leaders, and the scientific community to implement research in the context of the desired integration of services, sector wide approaches, harmonisation of activities, and long-term funding commitment. Thus, research areas such as social science or research into direct and indirect economic benefits of malaria eradication also need to be strengthened. With these drivers in place, and the development of the new tools we describe briefly here and in the other articles in this Supplement, it may be possible to fulfil the dream that malaria eradication can be achieved within the lifetime of young scientists just embarking on their careers, even in the most difficult areas where current tools/strategies have proven to be insufficient. The time course may be long, but to have a chance of realizing that dream, the commitment to starting those research and development efforts must begin now.

## Supporting Information

Text S1 **malERA governance bodies**
(0.05 MB DOC)

Text S2 **malERA launch meeting participants**
(0.06 MB DOC)

## Abbreviations

GMEP: Global Malaria Eradication Program
TPP: target product profile
