# Supplementary material for: A Research Agenda to Underpin Malaria Eradication
Source: PLoS Med. 2011 Jan 25;8(1):e1000406. doi: 10.1371/journal.pmed.1000406 (PMC3026687; doi:10.1371/journal.pmed.1000406)
Supplement: Text S1 — malERA governance bodies (0.05 MB DOC) [file pmed.1000406.s001.doc]

**Text S1: Malera Governance Bodies**

**MalERA Steering Committee**

**Pedro L. Alonso (chair)**

Barcelona Centre for International Health Research (CRESIB-Hospital Clínic, University of Barcelona), Barcelona (Spain) & Centro de Investigaçao em Saude da Manhiça, Manhiça (Mozambique)

**Myriam Arevalo-Herrera**

Immunology Institute, Universidad del Valle, Centro de Investigación Caucaseco, Cali (Colombia)

**Fred Binka**

INDEPTH Malaria Clinical Trials Alliance, Accra (Ghana)

**Chetan Chitnis**

International Centre for Engineering and Biotechnology, New Delhi (India)

**Frank Collins**

University of Notre Dame, Notre Dame (USA)

**Ogobara K. Doumbo**

Malaria Research and Training Centre, University of Bamako, Bamako (Mali)

**Brian Greenwood**

London School of Hygiene and Tropical Medicine, London (UK)

**B. Fenton Hall**

National Institute of Allergy and Infectious Diseases, Bethesda (USA)

**Kamini Mendis**

Global Malaria Program, World Health Organization, Geneva (Switzerland)

**Christopher V. Plowe**

Howard Hughes Medical Institute / University of Maryland School of Medicine, Maryland (USA)

**Mario Henry Rodríguez**

Instituto Nacional de Salud Pública, Cuernavaca (Mexico)

**Robert Sinden**

Imperial College, London (UK)

**Laurence Slutsker**

Centers for Disease Control and Prevention, Atlanta (USA)

**Marcel Tanner (co-chair)**

Swiss Tropical and Public Health Institute & University of Basel, Basel (Switzerland)

**MalERA International Advisory Committee**

**Peter Agre**

Malaria Research Institute, Bloomberg School of Public Health

Johns Hopkins University, Baltimore, (USA)

**Norman W. Baylor**

Office of Vaccines Research and Review, Center for Biologics Evaluation and Research, Rockville (USA)

**Maharaj K. Bhan**

Union Department of Biotechnology, Government of India, New Delhi (India)

**Zulfiqar Bhutta**

Aga Khan University Medical Center, Karachi (Pakistan)

**Joel Breman**

Fogarty International Center NIH, Bethesda (USA)

**Graham Brown**

Nossal Institute for Global Health, University of Melbourne, Melbourne (Australia)

**Lynda Chalker**

Medicines for Malaria Venture; AfricaMatters, London (UK)

**Tumani Corrah**

Medical Research Council Gambia, Banjul (The Gambia)

**Peter Doherty**

University of Melbourne, Melbourne (Australia)

**Walter R. Dowdle**

Task Force for Global Health, Decatur (USA)

**Richard Feacham**

University of California, Global Health Sciences, San Francisco (USA)

**François Gasse**

UNICEF, New York (USA)

**George Griffin**

St. Georges Medical School, London (UK)

**Donald A. Henderson**

Center for Biosecurity, University of Pittsburgh Medical Center, Baltimore (USA)

**Marie-Paule Kieny**

Initiative for Vaccine Research, WHO Geneva (Switzerland)

**Myron M. Levine (chair)**

Center for Vaccine Development, University of Maryland School of Medicine, Baltimore (USA)

**Tang Linhua**

National Institute of Parasitic Diseases, Centers for Disease Control, Shanghai (China)

**Pascoal Mocumbi**

European and Developing Countries Clinical Trials Partnership (EDCTP) Cape Town (South Africa)

**David Molyneux**

Liverpool School of Tropical Medicine, Liverpool (UK)

**Carlos Morel**

Center for Technological Development in Health of FIOCRUZ, Rio de Janeiro (Brazil)

**Hassan Mshinda**

Ministry of Science and Technology Tanzania, Dar es Salaam (Tanzania)

**José Nájera**

Independent, Formerly World Health Organization, Geneva (Switzerland)

**Ciro A. de Quadros**

Sabin Vaccine Institute, Washington DC (USA)

**Philippe Sansonetti**

Institute Pasteur, Paris (France)

**Peter Smith**

London School of Hygiene & Tropical Medicine, London (UK)

**Samba Sow**

Centre pour le Développement des Vaccins du Mali, Ministry of Health

Bamako (Mali)

**Yoshifumi Takeda**

Collaborative Research Center of Okayama University for Infectious Diseases in India at NICED, Kolkata (India)

**Roberto Tapia-Conyer**

Carso Health Institute, México D. F. (Mexico)

**Yeya T.Toure**

TDR WHO, Bamako (Mali)

**Rolf Zinkernagel**

Institute of Experimental Immunology, University of Zurich, Zurich (Switzerland)

**MalERA Leadership Council**

**Margaret Chan**
World Health Organization, Geneva (Switzerland)

**Awa Coll-Seck**
Roll Back Malaria Partnership, Geneva (Switzerland)

**Anthony S. Fauci**National Institute of Allergy and Infectious Diseases, Bethesda (USA)

**Mark Walport**
The Wellcome Trust, London (UK)

**Tadataka Yamada**
Global Health Program, Bill & Melinda Gates Foundation, Seattle (USA)
