# Supplementary material for: A Research Agenda to Underpin Malaria Eradication
Source: PLoS Med. 2011 Jan 25;8(1):e1000406. doi: 10.1371/journal.pmed.1000406 (PMC3026687; doi:10.1371/journal.pmed.1000406)
Supplement: Text S2 — malERA launch meeting participants (0.06 MB DOC) [file pmed.1000406.s002.doc]

**Launch meeting**

**20-21 Novembre 2008, Montreux, Switzerland**

**Salim Abdulla**

Ifakara Health Institute

Ifakara, Tanzania

**Edward Addai**

The Global Fund to Fight AIDS, Tuberculosis & Malaria

Geneva, Switzerland

**Pedro L. Alonso (chair)**

(Hospital Clínic, Universitat de Barcelona), Barcelona, Spain

Centro de Investigaçao em Saude da Manhiça, Mozambique

**Myriam Arevalo**

Immunology Institute for Medical Research

Cali, Colombia

**John W. Barnwell**

Centers for Disease Control and Prevention

Atlanta, U.S.A.

**Ian Bathurst**

Medicines for Malaria Venture

Geneva, Switzerland

**Fred Binka**

School of Public Health,

University of Ghana

Accra, Ghana

**Chetan Chitnis**

International Center for Genetic Engineering and

Biotechnology (ICGEB)

New Delhi, India

**Awa Coll-Seck**

Roll Back Malaria Partnership

Geneva, Switzerland

**Janice Culpepper**

Bill & Melinda Gates Foundation (BMGF)

Seattle, USA

**Hernando del Portillo**

Barcelona Center for International Health Research

Hospital Clínic-Universitat de Barcelona

Barcelona, Spain

**Stephen Duparc**

Medicines for Malaria Venture (MMV)

Geneva, Switzerland

**Domingo Gargallo**

Ferrer Group

Barcelona, Spain

**Brian Greenwood**

LSHTM

London, U.K.

**Pierre Guillet**

Independent

France

**Lee Hall**

National Institute of Allergy and Infectious Diseases (NIAID)

Bethesda, U.S.A.

**Janet Hemingway**

Liverpool School of Tropical Medicine

Liverpool, U.K.

**Sócrates Herrera**

Immunology Institute for Medical Research

Cali, Colombia

**Stephen Hoffman**

Sanaria

Rockville, U.S.A.

**Tony Holder**

National Institute for Medical Research

London, U.K.

**Marcelo Jacobs-Lorena**

Johns Hopkins Bloomberg School of Public Health

Baltimore, U.S.A.

**Peter Kremsner**

Tübingen University

Tübingen, Germany

**Myron M. Levine**

Center for Vaccine Development,

University of Maryland School of Medicine,

Baltimore, USA

**Christian Loucq**

PATH Malaria Vaccine Initiative

Washington D.C., U.S.A.

**Giancarlo Majori**

Instituto Superiore di Sanitá

Rome, Italy

**Dominique Mazier**

Université Pierre et Marie Curie

Paris, France

**Kamini Mendis**

World Health Organization (WHO)

Geneva, Switzerland

**Louis Miller**

National Institute of Health (NIH)

Bethesda, U.S.A.

**Jessica Milman**

Bill & Melinda Gates Foundation (BMGF)

Seattle, USA

**José Nájera**

Independent, formerly WHO

Geneva, Switzerland

**Hiro Nakatani**

World Health Organization (WHO)

Geneva, Switzerland

**Robert Newman**

Centers for Disease Control and Prevention

Atlanta, USA

**Francine Ntoumi**

Amanet Trust

Dar es Salaam, Tanzania

**Ayo Oduola**

World Health Organization (WHO)

Geneva, Switzerland

**Norma Padilla**

Universidad del Valle de Guatemala

Ciudad de Guatemala, Guatemala

**Christopher Plowe**

Howard Hughes Medical Institute / University of Maryland School of Medicine

Baltimore, USA

**Mario Henry Rodríguez López**

Instituto Nacional de Salud Pública

Cuernavaca, México

**Robert Sauerwein**

Radboud University Nijmegen Medical Center

Nijmegen, Holland

**David Schellenberg**

London School of Hygiene & Tropical Medicine

London, UK

**Louis Schofield**

The Walter and Eliza Hall Institute

Melbourne, Australia

**Robert Sinden**

Imperial College

London, U.K.

**Laurence Slutsker**

Centers for Disease Control and Prevention (CDC)

Atlanta, USA

**Sergio Spinaci**

World Health Organization (WHO)

Geneva, Switzerland

**Marcel Tanner**

Swiss Tropical Institute,

University of Basel,

Basel, Switzerland

**Awash Teklehaimanot**

Earth Institute at Columbia University

New York, U.S.A.

**Thomas Teuscher**

Roll Back Malaria Partnership

Geneva, Switzerland

**Tonya Villafana**

PATH Malaria Vaccine Initiative

Washington D.C., U.S.A.
